# Pan-Cancer Analyses Identify the CTC1-STN1-TEN1 Complex as a Protective Factor and Predictive Biomarker for Immune Checkpoint Blockade in Cancer

**DOI:** 10.3389/fgene.2022.859617

**Published:** 2022-03-16

**Authors:** Lishuai Wang, Tengfei Ma, Weijin Liu, Heping Li, Zhenhua Luo, Xuyang Feng

**Affiliations:** ^1^ Institute of Precision Medicine, The First Affiliated Hospital, Sun Yat-Sen University, Guangzhou, China; ^2^ Department of Medical Oncology, The First Affiliated Hospital, Sun Yat-Sen University, Guangzhou, China

**Keywords:** telomere, pan cancer, immune, prognosis, drug

## Abstract

The CTC1-STN1-TEN1 (CST) complex plays a crucial role in telomere replication and genome stability. However, the detailed mechanisms of CST regulation in cancer remain largely unknown. Here, we perform a comprehensive analysis of CST across 33 cancer types using multi-omic data from The Cancer Genome Atlas. In the genomic landscape, we identify CTC1/STN1 deletion and mutation and TEN1 amplification as the dominant alteration events. Expressions of CTC1 and STN1 are decreased in tumors compared to those in adjacent normal tissues. Clustering analysis based on CST expression reveals three cancer clusters displaying differences in survival, telomerase activity, cell proliferation, and genome stability. Interestingly, we find that CTC1 and STN1, but not TEN1, are co-expressed and associated with better survival. CTC1-STN1 is positively correlated with CD8 T cells and B cells and predicts a better response to immune checkpoint blockade in external datasets of cancer immunotherapy. Pathway analysis shows that MYC targets are negatively correlated with CTC1-STN1. We experimentally validated that knockout of CTC1 increased the mRNA level of c-MYC. Furthermore, CTC1 and STN1 are repressed by miRNAs and lncRNAs. Finally, by mining the connective map database, we discover a number of potential drugs that may target CST. In sum, this study illustrates CTC1-STN1 as a protective factor and provides broad molecular signatures for further functional and therapeutic studies of CST in cancer.

## Introduction

**FIGURE 1:**
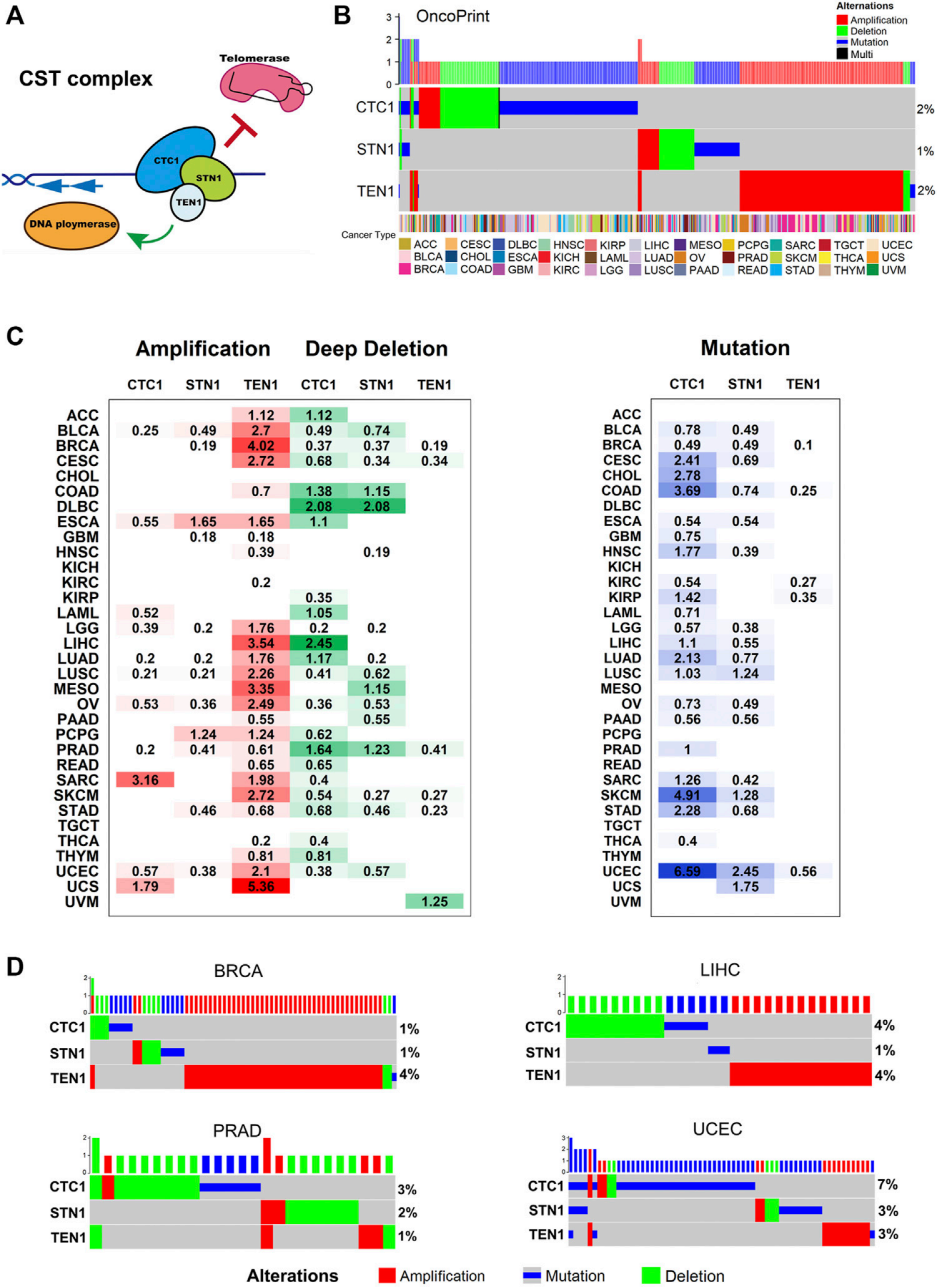
CST complex’s alteration in cancer: **(A)** schematic of the CST complex and its telomeric DNA binding and functions. **(B)** Landscape of genomic aberrations (non-silent mutation, amplification, and deep deletion) in the CST genes across cancer types. Each row represents a gene, and each column represents a sample. The frequencies are calculated over the entire cohort, with only altered samples plotted. The oncoprint plot displays the overall frequency of alterations in each gene in the right labels. The cancer type is shown in the color key at the bottom. **(C)** Distribution of SCNA (left) and mutation (right) and frequencies over cancer types. The degree of darkness is proportional to the frequency. **(D)** Genomic aberrations of the CST complex genes in BRCA, LIHC, PRAD, and UCEC. Each row represents a gene, and each column represents a patient sample. Green depicts deep deletion, red depicts amplification, and blue depicts mutation. Only samples with genomic alterations in the indicated genes are shown. Alteration rates per gene are displayed in the right labels.

Human CST is a heterotrimeric complex containing three components: CTC1, STN1, and TEN1, which plays multiple roles in maintaining telomere and genome integrity (Figure 1A) (Stewart et al., 2018; Nguyen et al., 2020; Lim and Cech, 2021). It directly binds to G-rich single-strand DNA, double-strand/single-strand DNA junctions, and some DNA secondary structures, such as G-quadruplexes (Chen et al., 2012; Bhattacharjee et al., 2017). CST is firstly identified as a telomere binding protein complex and functions in telomere replication and protection (Chen et al., 2012; Huang et al., 2012; Wang et al., 2012; Chen and Lingner, 2013; Feng et al., 2017; Ackerson et al., 2020). At telomeres, CST binds to the single-strand overhang region and removes telomerase from telomeres after G-stand synthesis (Chen et al., 2012; Feng et al., 2017). Then, it mediates the C-strand fill-in by interacting with some DNA polymerase (Huang et al., 2012; Wang et al., 2012; Feng et al., 2017; Ganduri and Lue, 2017; Feng et al., 2018). Besides its functions in telomere replication, CST also facilitates DNA replication in genome-wide by helping to restart the stalled replication forks (Stewart et al., 2012; Chastain et al., 2016; Wang et al., 2019; Schuck et al., 2021). Recent studies also reported that CST can mediate end protection at double-strand breaks, likely by using a similar strategy as the fill-in of the telomeric C-strand (Mirman et al., 2018). Supporting this observation, CST has been shown to promote ploy (ADP-ribose) polymerase inhibitor (PARPi) sensitivity in BRCA1-deficient cancer cells (Barazas et al., 2018; Mirman et al., 2018). Given its essential roles in replication and DNA repair, CST is known to be important for genome stability.

Mutations in CTC1 and STN1 are associated with pathologies referred to as “inherited telomere syndromes”, including dyskeratosis congenital and Coats Plus (Armanios and Blackburn, 2012; Simon et al., 2016; Shay and Wright, 2019). These syndromes are considered as a spectrum disorder showing a wide complex range of clinical symptoms, such as aplastic anemia, bone marrow failure, pulmonary fibrosis, gastrointestinal bleeding, retinal telangiectasias, and skin, hair, and nail changes and short telomeres (Armanios and Blackburn, 2012). Telomere shortening in these diseases induces genome instability that, in the absence of functional tumor suppressor genes, can contribute to tumorigenesis (Jafri et al., 2016; Martínez and Blasco, 2017). Therefore, CST’s crucial roles in both telomere maintenance and genome stability suggest its strong relevance to tumorigenesis. Recent studies also identify the essential functions of CST in cancer. Germline mutation of CTC1 was identified in patients with acute myeloid leukemia (Kim et al., 2020). CTC1 enhances the radioresistance of human melanoma cells by inhibiting telomere shortening and apoptosis (Luo et al., 2014). Downregulation of CTC1 by miR-376a induces telomere dysfunction and is associated with poor outcomes of patients in rectum adenocarcinoma (Liu et al., 2021). However, the role of CST in cancer is still not fully understood.

In this study, we observed gene alterations, expression, and epigenetic patterns of the members of CST. CTC1-STN1 was found to associate with a low level of telomerase activity, cell progression, genome instability, and better survival and better response to immune checkpoint inhibitors, demonstrating that CTC1-STN1 is a putative tumor suppressor. Experimentally, we confirmed that knockout of CTC1 decreased the c-MYC mRNA level. We also identified several chemical compounds that may modulate the expression of the CST complex. Finally, an online CST database (http://bioinfo-sysu.com/CSTDatabase/) was developed to provide rich datasets for hypothesis generation and experimental validation.

## Materials and Methods

### Somatic Mutation and Copy Number Alteration Analysis

We obtained The Cancer Genome Atlas (TCGA) pan-cancer somatic mutation data (MC3 MAF v0.2.8 file) from Genomic Data Commons (https://gdc.cancer.gov/about-data/publications/pancanatlas). The previously reported multiple filtering steps were used to eliminate artifacts and reduce false-positive rates (Thorsson et al., 2018). To include one of wga, native_wga_mix, and PASS, only the non-silent mutations were retained (the values in Variant_Classification column should be one of Frame_Shift_Del, Frame_Shift_Ins, In_Frame_Del, In_Frame_Ins, Missense_Mutation, Nonsense_Mutation, Nonstop_Mutation, Splice_Site, and Translation_Start_Site). Mutations calls were required to be called by two or more mutations callers (the values in NCALLERS column >1). The mutation hotpots were visualized using the MutationMapper tool in cBioPortal (https://www.cbioportal.org/mutation_mapper).

We obtained TCGA thresholded somatic copy-number alteration scores (SCNAs) (ISAR-corrected GISTIC2.0 all_thresholded.by_genes file) for 9,125 patient samples from Genome Data Commons. Genes assigned a positive value of +2 were considered as high-level amplification events, and genes with a negative value of −2 were considered as deep deletion events, as previously described (Stewart et al., 2018; Lim and Cech, 2021). OncoPrint plots of mutation and SCNA were generated by using the ComplexHeatmap R package.

### Pan-Cancer Analysis of CST Expression and CST Activity Score

The mRNA expression data from 10,304 samples of tumor and 719 samples of normal tissues across 33 cancer types were obtained from the TCGA database using the University of California Santa Cruz cancer genome browser (http://xena.ucsc.edu/). We collected transcriptome and clinical data and survival information. In this study, the log2-transformed RSEM was used for all mRNA analyses. We obtained 20 cancer types with a sufficient number of tumor-normal matched pairs (*n* ≥ 3). To assess the gene expression pattern of the CST complex, we used the Wilcox test to contrast the differences in CST gene expression levels between tumor and matched normal tissues and considered *p* value <0.05 as statistically significant.

To measure overall CST activity and CS activity in cancer, we derived a CST score and a CS score based on CST expression. First, the expression levels of CST genes were normalized within each cancer type according to previous publications (Korkut et al., 2018; Wang et al., 2018). Then, the CST score and CS score were obtained by calculating the first Z, normalizing the values for the CST genes. The mean across the genes was calculated for each sample to yield the CST score and CS score per sample.

### Scores for Telomerase Activity, Cell Stemness, and Genomic Instability

Telomerase activity was estimated from the expression of a 13-gene signature, named EXTEND score (Expression-based Telomerase ENzymatic activity Detection score) (Noureen et al., 2021). Cell stemness, an indicator of cell proliferation, was represented as the mRNAsi score calculated by using an innovative one-class logistic regression machine-learning algorithm (OCLR) (Malta et al., 2018). A previous pan-cancer study provided a list of genomic instability scores, including the mutation burden score, the homologous recombination deficiency (HRD) score, the SCNA burden score, the aneuploidy score, and the loss of heterozygosity (LOH) score (Knijnenburg et al., 2018). We assessed the level of the genetic instability score among the three expression clusters using the Kruskal test.

### Biological Pathway Analysis

The hallmark gene sets associated with the CST complex were identified by using the GSEA R package. We calculated the Spearman correlation coefficient between mRNA expression of all protein-coding genes and the CST complex. All the genes were ranked according to their correlation coefficients, and the pre-ranked gene lists were run against the MSigDB’s Hallmark Gene Set (HGS). All gene sets were annotated by their functional categories.

### Survival Analysis

The patients’ clinical data were retrieved from Genome Data Commons (https://gdc.cancer.gov/about-data/publications/pancanatlas). Survival analysis included 10,223 patients across 33 cancer types with complete transcriptome data and survival information. We assessed the correlation between the gene expression of the CST complex and the patient’s overall survival times. Kaplan–Meier survival curves of overall survival and the univariate Cox proportional hazards model were generated using the Survival and Survminer R packages. We used 1) The log-rank test to compare patient survival curves among the three clusters; 2) Within each cancer type, we determined the optimal cutpoint for the CTC1-STN1 score (CS score) and TEN1 expression using the maximally selected rank statistics from the maxstat R package. Then, the statistical significance of survival differences between the low and high groups was determined using the log-rank test; and 3) the univariate Cox proportional hazards model to assess the correlation between CST expression and the patient’s overall survival time.

### Analysis of Response to Immune Checkpoint Blockade

Gene expression data and overall survival data were obtained from four independent cancer datasets, including metastatic melanoma (GSE78220, GSE91061, and GSE100797) and metastatic urothelial cancer (IMvigor). The CS score of each sample was calculated in each dataset. Kaplan–Meier survival curves of overall survival were generated using the Survival and Survminer R packages. In metastatic melanoma, the correlation between CS score and PD-L1 expression in each dataset was calculated by linear regression in R. In the IMvigor dataset, we analyzed the correlation between CS score and TGFBR2 expression.

### Tumor Immune Infiltration Analysis

The robust estimation data of immune infiltration levels for TCGA using six algorithms (CIBERSORT, EPIC, MCPCOUNTER, QUANTISEQ, TIMER, and XCELL) were downloaded from the Tumor Immune Estimation Resource database (TIMER 2.0, http://timer.cistrome.org/). We analyzed the correlation between CS score and immune cell infiltration levels within each cancer type. The correlations of CS score with immune infiltration were evaluated by tumor purity-adjusted partial Spearman’s correlation and statistical significance.

### Analysis of Tumor Immune Evasion

TIDE (tumor immune dysfunction and exclusion) is a computational method to model two primary mechanisms of tumor immune evasion: T cell dysfunction and T cell exclusion. The TIDE score can predict immune checkpoint blockade (ICB) clinical response based on pre-treatment tumor profiles. The TIDE score of each sample in the TCGA cohort was analyzed by using the web application (http://tide.dfci.harvard.edu). We further calculated Spearman correlation coefficients between CST score, CS score, Ten1 expression, and TIDE score in each cancer type.

### DNA Methylation Analysis

TCGA DNA methylation data files were downloaded from Genomic Data Commons. We mapped the Illumina methylation array probes to individual genes using the Illumina Human Methylation 450 k R annotation data package. We retained those probed mapped to the promoter region. For genes with multiple probes, mean beta values were used. To estimate the overall methylation level, we then calculated the median beta value for CST genes in each sample. To examine the regulation of CST expression by DNA methylation, we calculated the Spearman correlation between the DNA methylation beta value and mRNA expression for each gene.

### LncRNA Analysis

A recent pan-cancer study reconstructed lncRNA regulatory networks (lncNETs) using the molecular tumors of TCGA (Feng et al., 2017). We retrieved a list of lncRNA that is associated with CST expression. Next, we retained those lncRNAs which had a Spearman correlation coefficient < −0.15 and *p* < 0.05 with CTC1 and STN1. The gene sets associated with that lncRNA were identified by using the GSEA R package.

### miRNA Analysis

The batched-corrected, normalized miRNA expression data (pancanMiRs_EBadjOnProtocolPlatformWithoutRepsWithUnCorrectMiRs_08_04_16. xena) were downloaded from the TCGA database using the University of California Santa Cruz cancer genome browser (http://xena.ucsc.edu/). We calculated Spearman correlation coefficients between expression for miRNA mature strands and mRNA expression levels of the three CST genes in individual cancer types. We filtered the result by requiring correlations to have a coefficient < −0.2 and *p* > 0.05. Then, we filtered by retaining miRNA with experimentally validated miRNA–mRNA interactions (from TargetScan 7.2 database and miRDB database).

### Connectivity Map Analysis

Connectivity Map (CMap, https://clue.io/) is a chemical genomics database that collects gene-expression profiles from cultured human cells treated with small molecules (Huang et al., 2012). In each cancer type, there is a list of top 150 positively or negatively correlated genes associated with CST score, CS score, and TEN1 expression level. Compounds with enrichment score >90 or < −90 in at least 17 cancer types (>1/2 of all cancer types) are significantly positive or negative compounds, respectively.

### Database Construction

The CST database is a resource tool open to all users. It is constructed in the standard MVC (Model-View-Controller) pattern, which consists of a server side and a client side. The outputs of the database are plots and tables. All plots are created by R (version R ×64 4.0.3).

### Graph Plotting

Heatmaps for gene expression data and GSEA results were depicted using the pheatmap R package. The OncoPrint plots of mutation and SCNA were generated using the ComplexHeatmap R package. The differences among the three clusters were plotted as a boxplot by using the ggplot2 R package. Cytoscape software is a free open-source platform that enables biological network analysis and two-dimensional visualization. The connective network among CTC1, STN1, lncRNA, and pathways was visualized using Cytoscape (version 3.7.2). The Kaplan–Meier survival curves of overall survival were plotted using the survminer and survival R packages. Cox regression was performed using the survival R package and visualized using the ggplot2 R package. The miRNA-CST regulation and lncRNA-CST regulation were plotted as heatmaps by using the pheatmap R package.

### Cell Culture

CTC1 and TEN1 conditional knockout HCT116 cells were cultured in McCoy’s 5A Medium (modified) supplemented with 10% FBS, with antibiotics as previously described (Feng et al., 2017; Feng et al., 2018). Tamoxifen (Sigma, H7904) was added to 10 nM for 7 days to induce Cre expression to disrupt the *CTC1* or *TEN1* gene.

### Quantitative Real-Time PCR

The total RNA was extracted from cells using the Trizol Reagent (Invitrogen) according to the manufacturer’s protocol. To generate cDNA, total RNA by reverse transcription using the PrimeScript™ RT reagent Kit with gDNA Eraser (TaKaRa) was determined. cDNA was analyzed by qPCR with QuantStudio (Thermo Fisher) using PowerUp™ SYBR™ Green Master Mix (Thermo Fisher). The relative expression levels of the target genes were determined by the 2^−ΔΔCt^ method.

### Primers for qPCR Were as Follows

CTC1 forward 5′−CTA​GAC​TCC​TAT​CCT​ATT​CGG​GA−3′ and reverse 5′−ACA​CAC​TCC​AGG​TCG​CAT​C−3′, TEN1 forward 5′−GGC​CAA​GTT​CCT​GAT​GGG−3′ and reverse 5′− CAG​TGT​TAC​TCT​GGA​CTG​AAT​CAT−3′, C-MYC forward 5′− CGT​CTC​CAC​ACA​TCA​GCA​CAA−3′ and reverse 5′− CAC​TGT​CCA​ACT​TGA​CCC​TCT​TG−3′.

## Results

### Somatic Alteration Analysis Identifies CTC1/STN1 Deletion and Mutation and TEN1 Amplification as Major Alteration Events

To investigate the genomic characteristics of the CST complex in cancers, we analyzed the mutation frequency and SCNA of patients across 33 cancer types in the pan-cancer cohort. Genetic alterations include gene mutations (truncating and missense) and SCNA (amplification and deep deletion). The proportion of CST alterations was relatively low; about 4.79% of tumors had at least one type of the alterations. The OncoPrint shows a panoramic view of CST genomic aberrations (Figure 1B). The overall genetic alteration of CST genes ranged from 1 to 2%. Within the CST complex, the predominant alteration pattern varied for each gene. The alterations of *CTC1* and *STN1* were dominated by mutations and deletions, while *TEN1* was more common by amplifications (Figures 1B,C). The lollipop plots show the mutation patterns and some potential recurrent hotspot mutations in CST (Supplementary Figure S1A). We found two significant mutation sites, which were missense mutations of *STN1* (H317Y) and *TEN1* (R119P/Q/W) (Supplementary Figure S1A). Examining the expression level of the corresponding genes among different alteration groups, we found that the amplification group displayed the highest expression, while the deletion group showed the lowest expression in all the three genes (Supplementary Figure S1B).

When examining the genetic alteration status of the CST complex across 33 cancer types, we found that various cancer types have different patterns of genetic alterations. A high-amplification frequency of *TEN1* was observed in breast invasive cancer (BRCA), liver hepatocellular carcinoma (LIHC), mesothelioma (MESO), and uterine carcinosarcoma (UCS), while a relatively high deletion rate of *CTC1* and *STN1* was found in colon adenocarcinoma (COAD), lymphoid neoplasm diffuse large B-cell lymphoma (DLBC), and LIHC and prostate adenocarcinoma (PRAD) (Figure 1C). CTC1 mutation predominately occurred in uterine corpus endometrial carcinoma (UCEC) (6.59%), skin cutaneous melanoma (SKCM) (4.91%), and COAD (3.69%) (Figure 1C). Some cancer types were found with both alterations of *TEN1* amplification/*CTC1* deletion (LIHC) or both of *CTC1*/*STN1* deletions (PRAD) or all of the *TEN1* amplifications and *CTC*1/*STN1* deletions (BRCA and UCEC). To investigate whether the two/three alternations simultaneously existed in these cancers, we did OncoPrint for each cancer type. However, we found few overlaps of different alternation types in one patient (Figure 1D). Particularly, in BRCA, *TEN1* amplification was the most frequent type of genetic alteration. In LIHC, the proportion of *CTC1* deletion or mutation and *TEN1* amplification was 4%. The alteration pattern of the CST complex in PRAD is mainly the deletion of CTC1 and STN1, but CTC1 mutations are more common in UCEC (Figure 1D). These data illustrate a diverse alteration pattern for the CST complex, with mutation and deletion for *CTC1* and *STN1*, and amplification for *TEN1* as major events. The major events are not simultaneous in any cancer type, indicating that the individual component of the CST complex has different expression profiles and functions in cancer.

### Gene Expression Analysis Reveals Three Patient Clusters Exhibiting Differences in Survival, Telomerase Activity, Cancer Stemness, and Genome Stability

**FIGURE 2:**
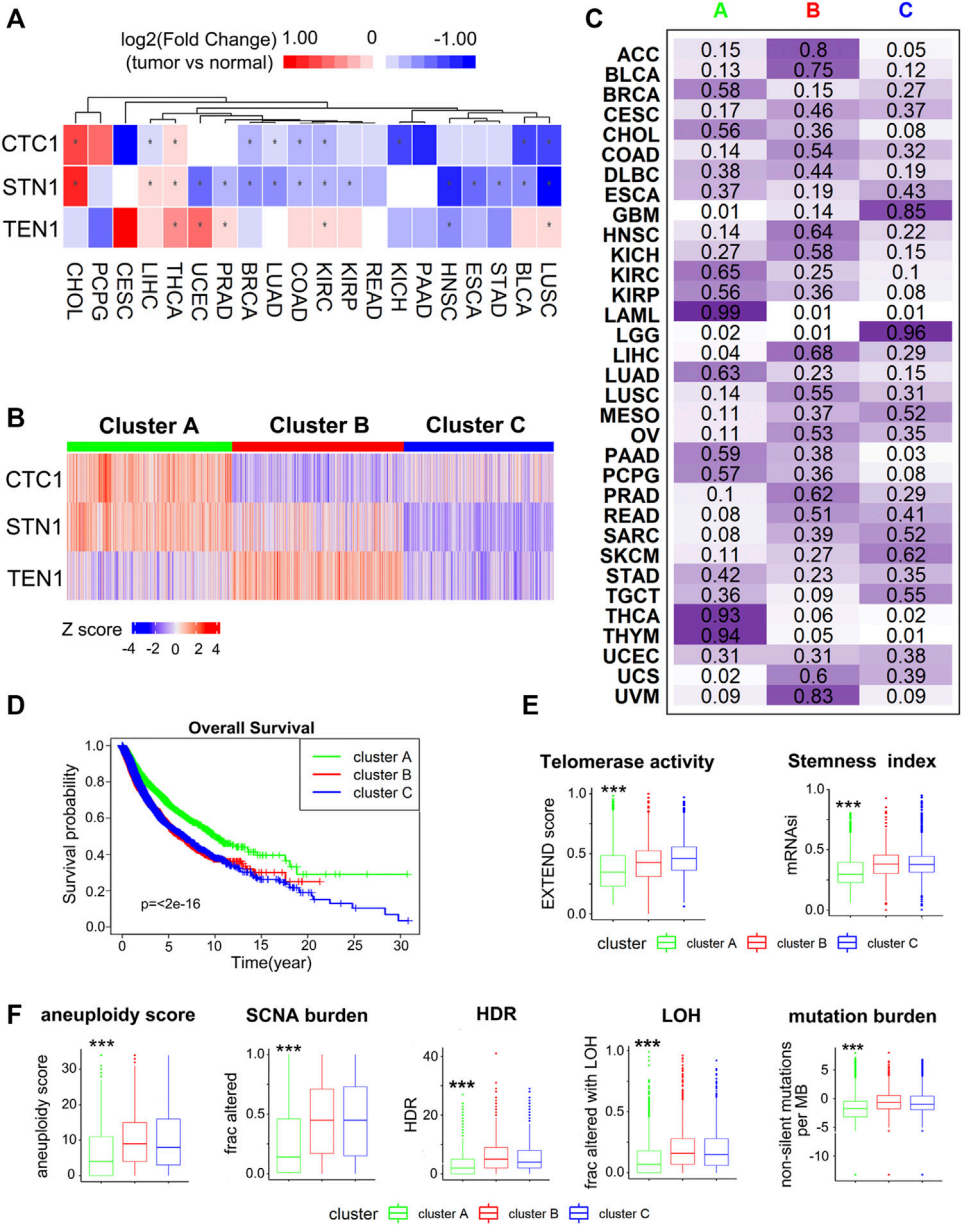
Expression landscape of the CST complex: **(A)** differential expression of CST between the tumor and adjacent normal tissue for 20 cancer types that have more than three adjacent normal samples. In the heatmap, red indicates high expression in the tumor, and blue indicates low expression, *p < 0.05. **(B)** Unsupervised clustering of CST gene expression reveals three distinct clusters marked by a different color in the top box. Each row represents a gene, and each column is a patient. Red indicates high expression, and blue indicates low expression. The expression data were normalized by z-score normalization for each row. Unsupervised clustering used k-means clustering. **(C)** Sample distribution in the three clusters. Each row represents a cancer type, and each column represents a cluster. The number and the color intensity in each box show the percentage of samples classified in the corresponding cluster. **(D)** Kaplan–Meier curves of overall survival in three clusters. Statistical analysis was performed using the log-rank test. **(E)** Boxplots show the telomerase activity (left) and stemness index (right) in three clusters. **(F)** Boxplots display the levels of a list of genome instability scores among the three clusters, including aneuploidy score, SCNA burden, HRD, homologous recombination deficiency, LOH, loss of heterozygosity, and mutation burden. The differences among three clusters were tested by the Wilcoxon test, ***p < 0.001.

When comparing the expression of CST between tumor and paired adjacent normal samples, we found that CTC1 and STN1 were largely downregulated, whereas TEN1 was upregulated in tumors (Figure 2A). CTC1 and STN1 presented consistently lower expression in tumor samples for the majority of cancers, including bladder urothelial carcinoma (BLCA), BRCA, COAD, kidney chromophobe (KICH), kidney renal clear cell carcinoma (KIRC), lung adenocarcinoma (LUAD), and lung squamous cell carcinoma (LUSC) (*p* < 0.05). In contrast, TEN1 was upregulated in LUSC, KIRC, PRAD, thyroid carcinoma (THCA), and UCEC (*p* < 0.05) (Figure 2A).

To gain a comprehensive view of CST expression, unsupervised consensus clustering was performed on patient samples across 33 cancer types based on the expression of the CST complex using the K-means clustering method. Three distinct clusters emerged from cluster analysis, with cluster A displaying the highest expression of CTC1 and STN1 and low expression of TEN1, while cluster B had the most abundant expression of TEN1 (Figure 2B). Analyzing the distribution of samples, we found that acute myeloid leukemia (LAML) (99%), THCA (93%), and thymoma (THYM) (94%) were mainly located in cluster A, whereas glioblastoma multiforme (GBM), brain lower grade glioma (LGG), LIHC, and UCS were nearly depleted in this cluster (the proportion of samples was less than 5%) (Figure 2C). Adrenocortical carcinoma (ACC) (80%), BLCA (75%), and uveal melanoma (UVM) (83%) were largely located in cluster B. GBM (85%) and LGG (96%) are common central nervous system tumors, predominately resided in cluster C (Figure 2C). The samples of some cancer types were almost equally distributed among the three clusters, including DLBC, esophageal carcinoma (ESCA), stomach adenocarcinoma (STAD), and UCEC (Figure 2C). These data indicate that there is a dysregulated expression profile of the CST complex in a cancer-type-dependent manner.

The CST complex has been demonstrated to regulate telomerase, cell growth, and genome integrity. To assess the functionality of the expression pattern, we examined a group of scores measuring telomerase activity (represented as EXTEND score), cell stemness (an indicator for cancer progression), and genome instability (scores for aneuploidy score, SCNA burden, HDR, LOH, and mutation burden). We first found that three clusters showed different survival rates (Figure 2D). Cluster A, which had the highest expression of CTC1 and STN1, showed the best survival (Figure 2D). Additionally, cluster A had the lowest levels of scores for telomerase activity, stemness, and genome instability (Figures 2E,F). Collectively, these data not only confirm that the CST complex is a terminator of telomerase and that it maintains genome integrity but also implicate that CTC1 and STN1 can still execute these functions when TEN1 is low.

### CTC1 and STN1, but Not TEN1, Are Favorable Predictors of Survival

Having established an initial link between the CST complex and survival, we further investigated this link in greater detail. To measure the activity of the CST complex, we derived the CST score (based on mRNA expression of three CST genes) for each cancer type. We also derived the CS score (based on mRNA expression of CTC1 and STN1) as CTC1-STN1 had distinct expression profiles when compared to TEN1.

**FIGURE 3:**
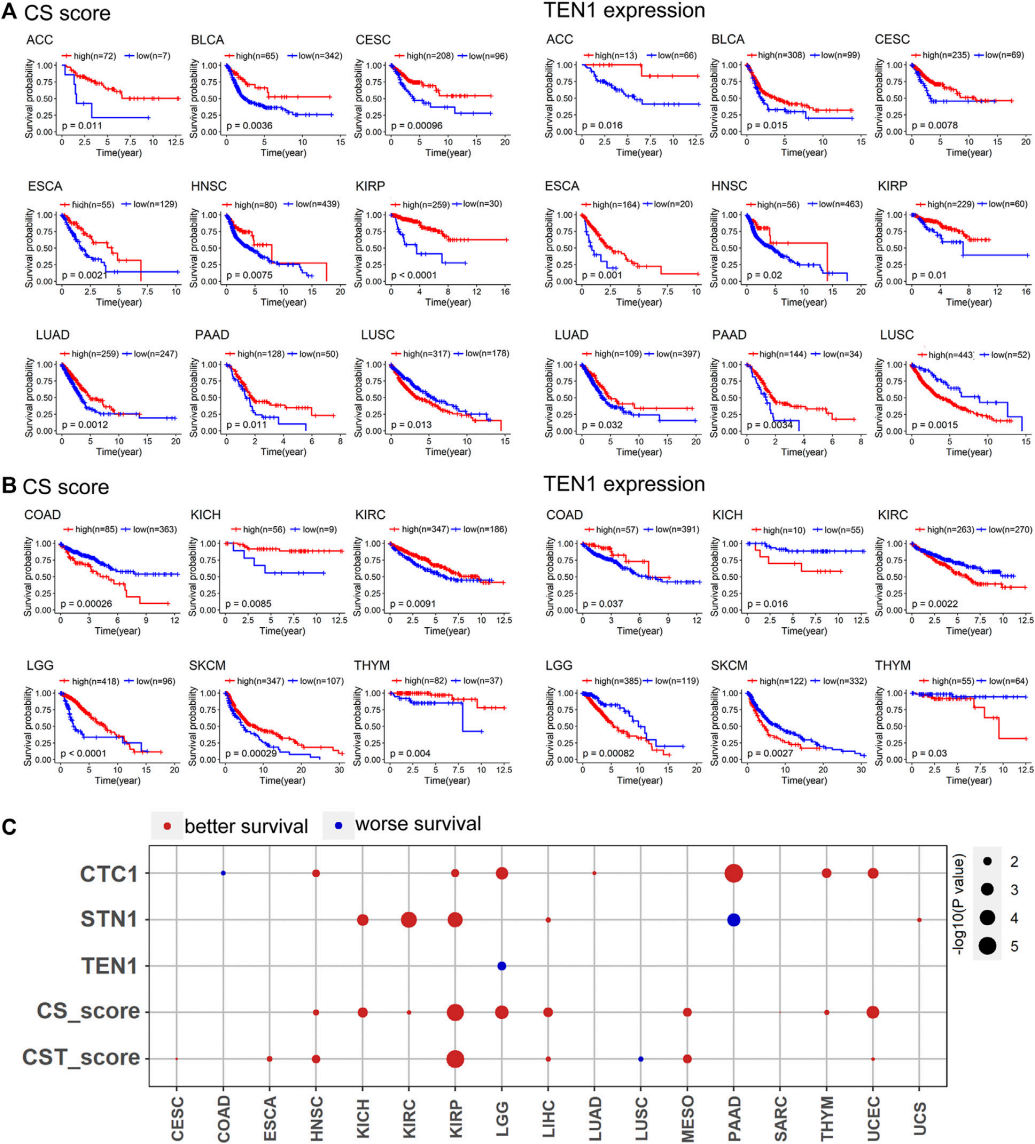
CTC1 and STN1 predict better survival: **(A)** CS score and TEN1 expression were consistent in predicting the prognosis of nine cancer types. A high CS score and high TEN1 expression were positively correlated with overall survival, except for LUSC. Statistical analysis was performed using the log-rank test. **(B)** CS score and TEN1 gene expression were not consistent in predicting the prognosis of six cancer types, including COAD, KICH, KIRC, LGG, SKCM, and THYM. Statistical analysis was performed using the log-rank test. **(C)** Summary of COX regression correlation of CST with survival. Red indicates better survival, and blue indicates worse survival. Only significant dots with *p* value <0.05 are shown. The size of the dot is proportional to the –log10(*p* value).

To obtain the predictive value of the CS score and TEN1 expression in overall survival, we performed Kaplan–Meier survival curve analysis by grouping the patients into the high and low groups based on optimal cutoff values of the CS score and TEN1, respectively. The survival analyses reveal two cancer groups in which both CS score and TEN1 are significantly associated with survival (Figures 3A,B). The first cancer group contained nine cancer types showing a consistent survival pattern for CS score and TEN1 (Figure 3A). In contrast, the second cancer group had six cancer types exhibiting an opposed survival pattern for CS score and TEN1 (Figure 3B). Among 15 cancer types, the CS score predicted better survival in 13 cancer types including ACC, BLCA, cervical squamous cell carcinoma and endocervical adenocarcinoma (CESC), ESCA, head and neck squamous cell carcinoma (HNSC), kidney renal papillary cell carcinoma (KIRP), LUAD, pancreatic adenocarcinoma (PAAD), KICH, KIRC, LGG, SKCM, and THYM. However, TEN1 was associated with better survival in nine cancer types and with worse survival in six cancer types (Figures 3A,B). To confirm these observations, we thus performed Cox regression analysis. Cox regression revealed that CTC1, STN1, the CS score, and the CST score were associated with better survival in at least four cancer types, while TEN1 was associated with worse survival only in LGG (Figure 3C). Taking all these data together with data in Figure 2A, we conclude that CTC1 and STN1, but not TEN1, may prevent tumor progression and protective factors for survival in many cancer types.

### CTC1 May Be a Negative Regulator of c-MYC

**FIGURE 4:**
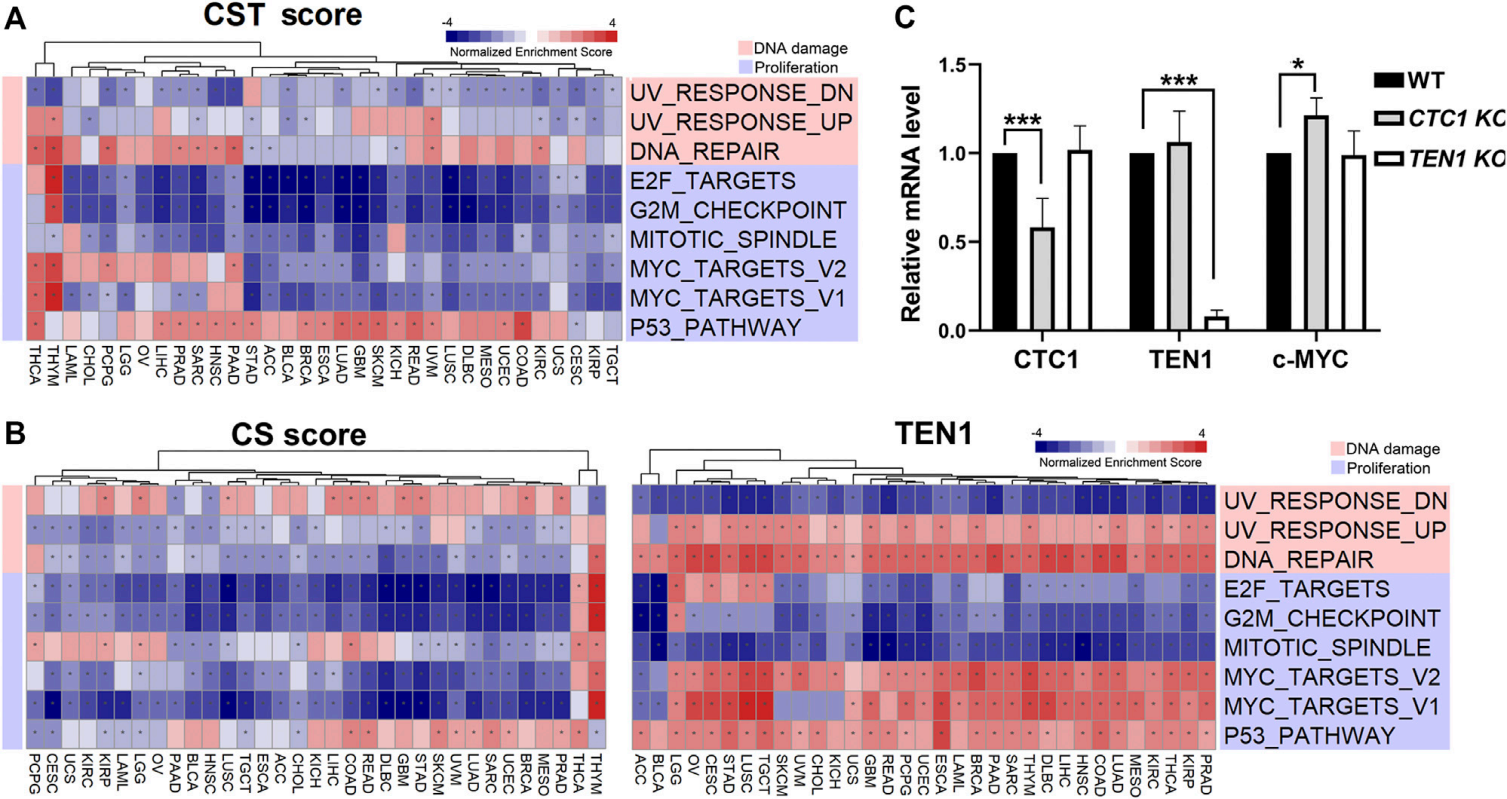
CST-correlated pathways: **(A)** heatmap showing normalized enrichment score of significant hallmark sets of CST score. **(B)** Heatmap showing normalized enrichment score of significant hallmark sets of CS score (left) and TEN1 expression (right). Each column represents a cancer type, and each row represents a hallmark set. Red represents positive normalized enrichment scores, and blue represents negative enrichment scores. The “*” symbol in cells indicates that enrichment is statistically significant. Unsupervised clustering used the Euclidean distance metric with complete linkage. The functions of the significant hallmark sets are annotated in different colors. **(C)** Quantitative PCR was carried out to determine the mRNA levels of endogenous CTC1, TEN1, and c-MYC in the wild type, CTC1, or TEN1 conditional knockout HCT116 cells. The differences were tested by Student’s *t* test, ***p < 0.001.

To understand the underlying mechanisms by which the CST complex regulates survival, cell proliferation, and genome integrity, we performed gene set enrichment analysis using the CST score, CS score, and TEN1 expression. This analysis identified several significant gene sets (Supplementary Figures S2A,B). Among them, the CST and CS score were negative, whereas TEN1 was positively associated with DNA repair, upregulated genes in response to UV (UV_RESPONSE_UP), and MYC targets (Figures 4A,B). Experimentally, we confirmed that knockout of CTC1, but not TEN1, increased the c-MYC mRNA level (Figure 4C). Altogether, these data indicated that CTC1 may be a negative regulator of c-MYC.

### CTC1 and STN1 Predict Better Response to Immune Checkpoint Blockade

**FIGURE 5:**
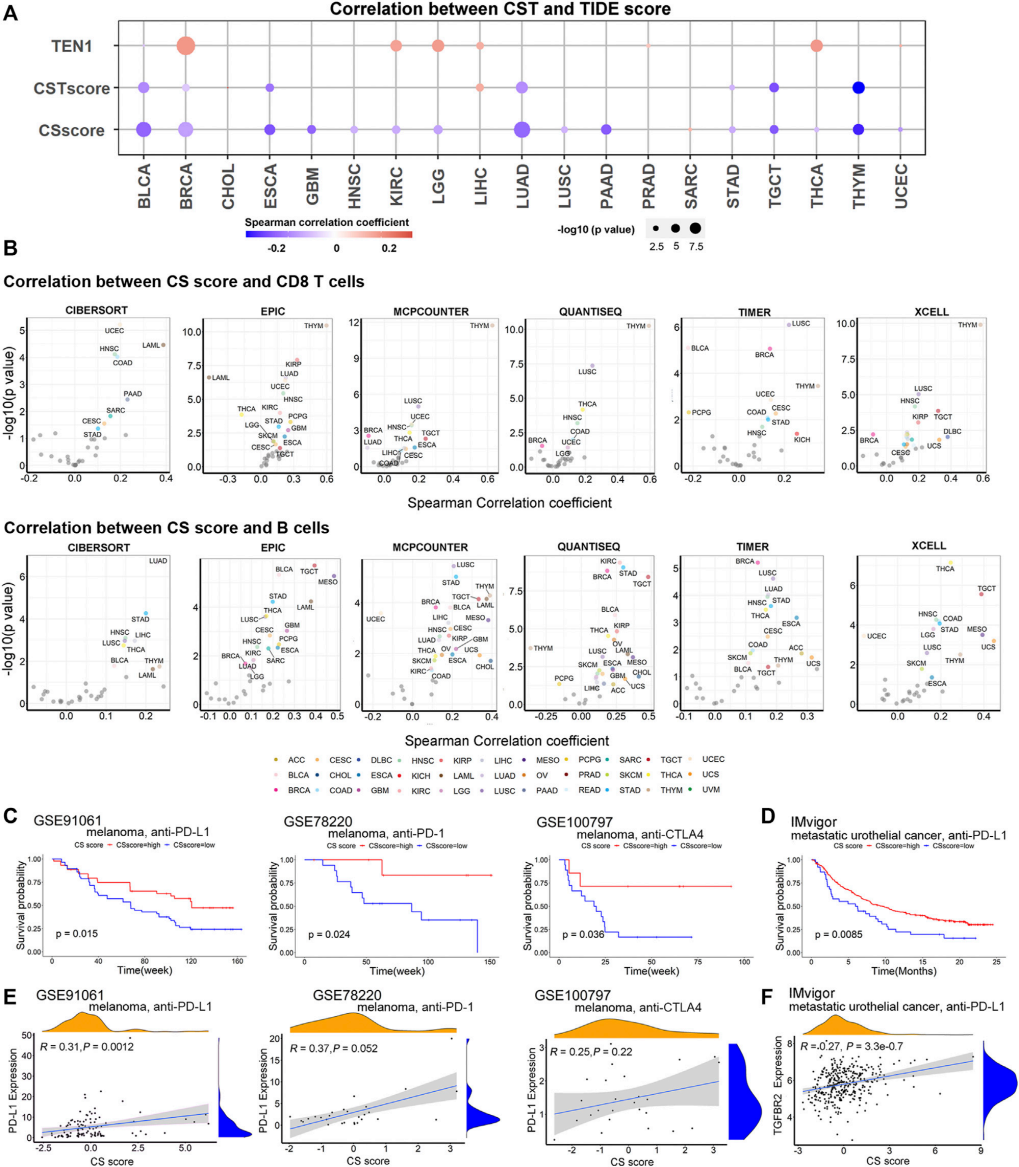
CTC1 and STN1 are associated with better response to immune therapy: **(A)** TIDE score reflects T cell dysfunction and exclusion and predicts cancer immunotherapy response. Bubble plots showing correlation between CST score, CS score, TEN1 expression, and TIDE score. Red represents positive correlation, and blue represents negative correlation. **(B)** Volcano plots showing correlation between CS score and tumor-infiltrating immune cells in each cancer type estimated by Tumor Immune Estimation Resource (TIMER). Cancers with a significant positive and negative association between CS score and antitumor immune cells (CD8 T cells and B cells) after using tumor purity-corrected partial Spearman correlation analysis are highlighted in different colors, respectively. **(C,D)** Kaplan–Meier survival curves showing a high CS score can reflect improved overall survival of metastatic melanoma **(C)** and metastatic urothelial cancer **(D)**, suggesting CS score as a potential predictor of response to immunotherapy. **(E)** Correlation plots showing correlation between CS score and expression of PD-L1 in metastatic melanoma patients treated with immune checkpoint blockade therapy, including anti-PD-1, anti-PD-L1, and anti-CTLA4 antibodies. **(F)** Correlation plots showing that CS score is positively associated with expression of TGFBR2 in metastatic urothelial cancer treated with the anti-PD-L1 agent. *R*, Spearman correlation coefficient; *P*, *p* value of Spearman correlation.

We noticed that CST had both positive and negative correlations with several immune-related gene sets dependent on the cancer type (Supplementary Figures S2). To further study how CST connects with cancer immunity, we calculated the correlation between CST and tumor immune dysfunction and exclusion (TIDE) score. TIDE score is a measurement of tumor immune escape, reflecting T cell dysfunction and exclusion and predicting cancer immunotherapy response (Jiang et al., 2018). We observed that CTC1 and STN1 (i.e., the CS score) were negatively associated with the TIDE score, suggesting that the CS score predicted a better response to immunotherapy (Figure 5A). Using six immune cell estimation methods, we found that the CS score was positively correlated with CD8 T cells and B cells in several TCGA cancer types (Figure 5B). CD8 T cells and B cells are important biomarkers for immune checkpoint blockade (Riaz et al., 2017; Helmink et al., 2020). The abundance of CD8 T cells and B cells was positively correlated with a favorable response to immune checkpoint blockade. Thus, all these data indicate that CTC1-STN1 might serve as a potential predictive biomarker for immune checkpoint blockade.

To confirm the predictive value of CTC1-STN1, we curated three datasets of expression data for melanoma patients treated with anti-PD-1, anti-PD-L1, or anti-CTLA4 and one dataset of expression data for metastatic urothelial cancer patients treated with anti-PD-L1 (Hugo et al., 2016; Riaz et al., 2017; Lauss et al., 2017; Mariathasan et al., 2018). For all these datasets, the CS score predicted a better response. Patients with a high CS score showed better survival (Figure 5C,D). Supporting this observation, we also found that CS score was positively correlated with PD-L1 in melanoma and with TGF-beta receptor TGFBR2 in metastatic urothelial cancer (Figures 5E,F). PD-L1 and TGFBR2 had been shown to be higher in the good responder group (Patel and Kurzrock, 2015; Mariathasan et al., 2018). Taken all these data, we conclude that CTC1 and STN1 predict the better response to immune therapy.

### miRNAs and lncRNAs, but not DNA Methylation, Suppress CTC1 and STN1 Expression

**FIGURE 6:**
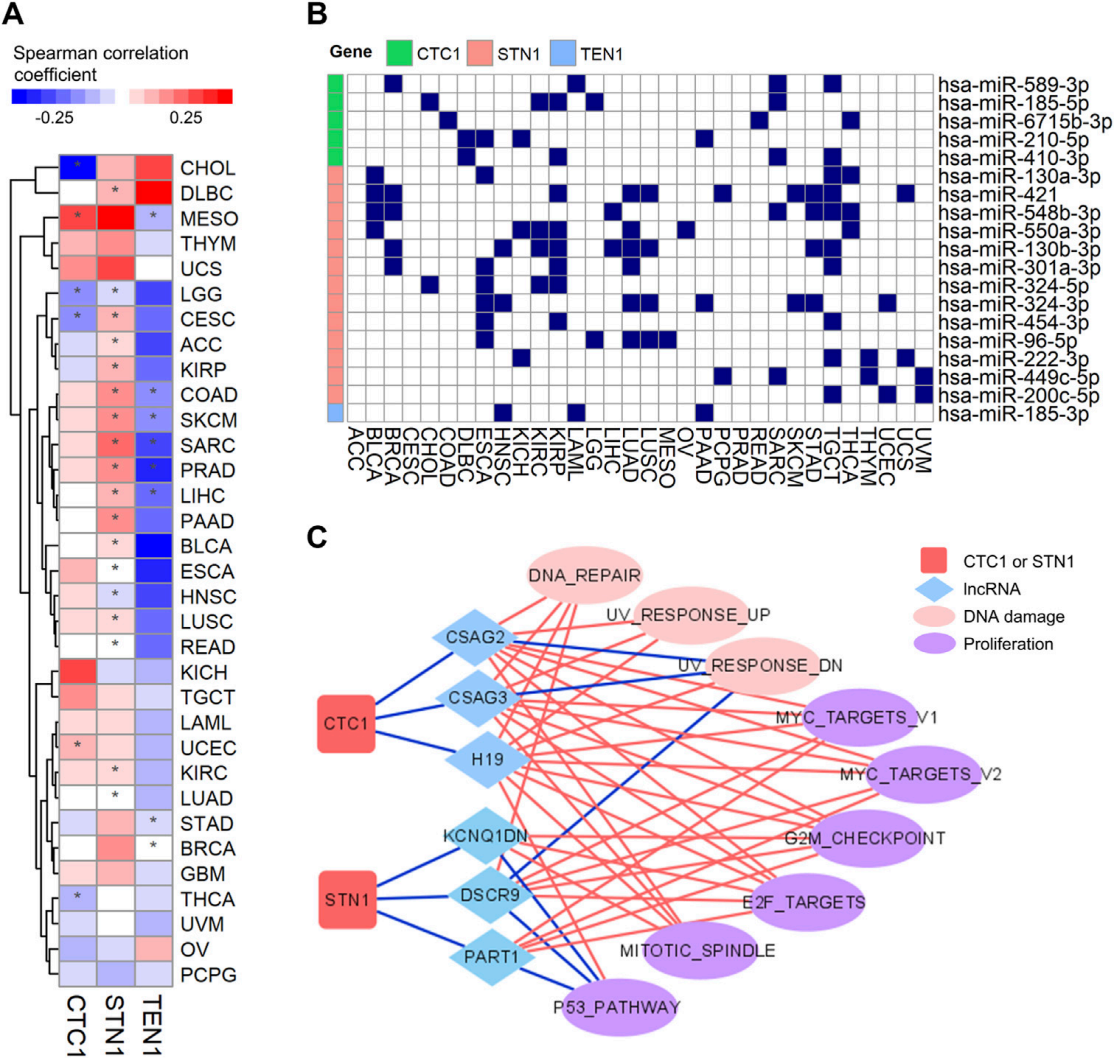
Regulations of expression of the CST complex by DNA methylation, lncRNA, and miRNA: **(A)** heatmap shows Spearman correlation coefficient between CST expression and DNA methylation level (beta value) within promoters in each cancer type. Red indicates positive correlation, and blue indicates negative correlation. The * symbol indicates a statistically significant (*p < 0.05). **(B)** Graph showing miRNAs and their CST targets in multiple tumor types. The blue rectangle indicates that miRNA interacts with its targets. **(C)** Connective network among CTC1, STN1, lncRNA, and pathways. The red line indicates positive connection, while the blue line indicates negative connection.

To investigate mechanisms that were responsible for dysregulated expression of the CST complex, we focused on DNA methylation, miRNAs, and lncRNAs as they are key regulators of gene expression. Surprisingly, Spearman correlation analyses between CST expression and the corresponding methylation level showed that the DNA methylation level of TEN1 was negatively correlated with its mRNA expression, while those of CTC1 and STN1 were positively correlated (Figure 6A). This positive correlation data suggest that DNA methylation was not responsible for the downregulation of CTC1 and STN1 expression in tumor samples.

Therefore, we tested whether non-coding RNAs could be an important regulator. Interestingly, by mining the miRNA-target interaction database and analyzing the correlation between miRNA and CST expression, we found that CTC1 and STN1, but not TEN1, could be repressed by multiple miRNAs in various cancer types (Figure 6B). In addition, we observed that some lncRNAs negatively associated with CTC1 and STN1 expression. Remarkably, those lncRNAs positively associated with those pathways had negative connections with CTC1 and STN1 (Figure 6C). For example, the negatively correlated pathway of CTC1/STN1 and MYC_TARGETS_V1 (Figure 4B) was positively correlated with CTC1/STN1-associated lncRNAs including CSAG2, CSAG3, H19, DSCR9, and PART1 (Figure 6C), suggesting that lncRNAs might enhance MYC expression by downregulating CTC1 and STN1. Collectively, these findings suggest that non-coding RNAs might be responsible for the suppression of CTC1 and STN1 in cancer.

### Connectivity Map Analysis Identifies Compounds Targeting CST Signatures

**FIGURE 7:**
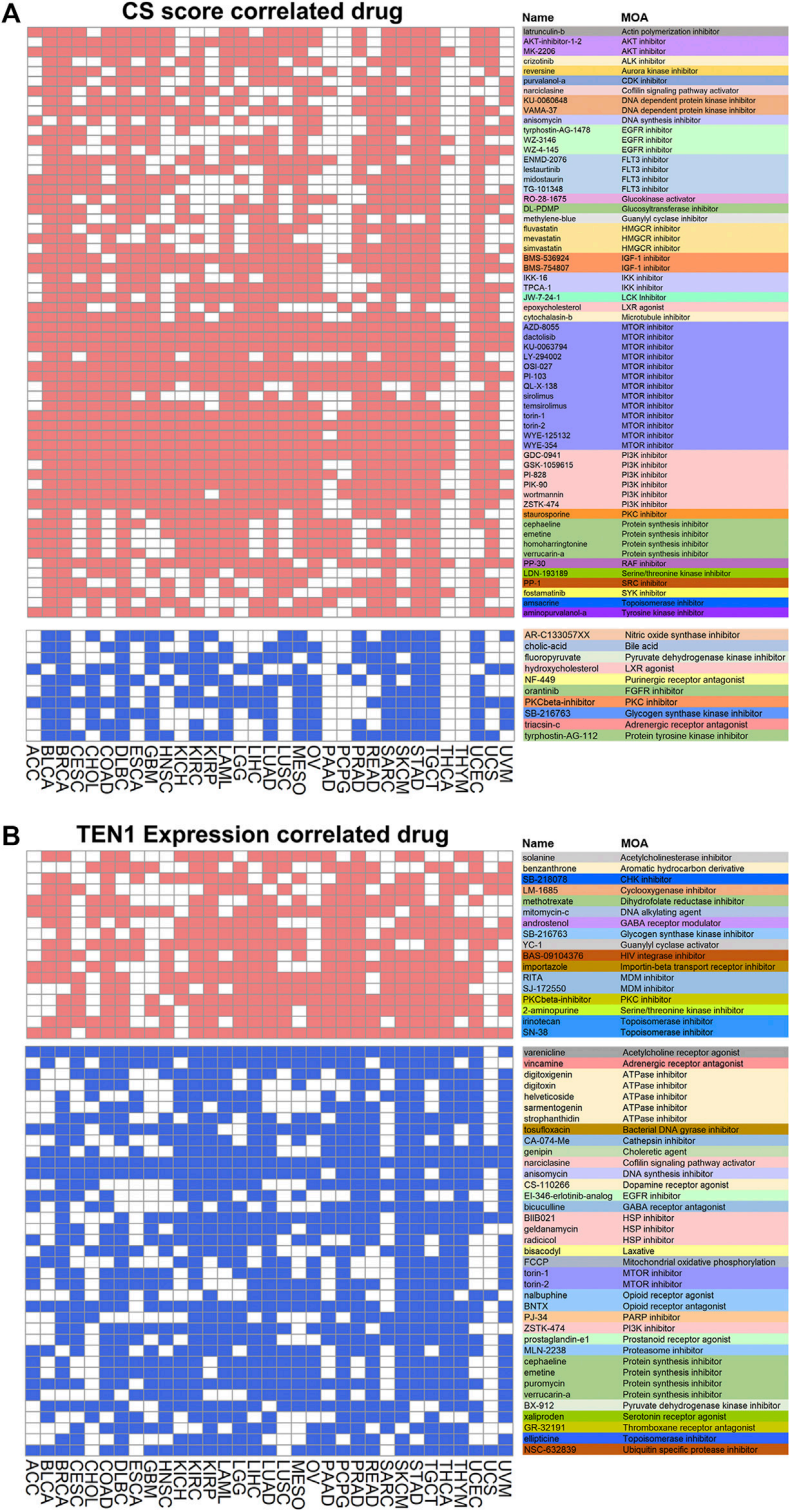
Correlation of CST with drugs: **(A,B)** heatmaps show drugs related to CS score **(A)** and TEN1 expression **(B)**. The heatmap shows positive (red) and negative (blue) correlation of each compound from the connectivity map for each cancer type. A significant correlation is defined with enrichment score > 90 or < −90. The functions of the drug are annotated in different colors.

CST has been reported to promote PARP inhibitor sensitivity in BRCA1-deficient cancer cells, highlighting that it can be a potential therapeutic target. Thus, we searched for chemical compounds that may modulate CST expression by connectivity map analysis. CS score was used since CTC1 and STN1 were co-expressed in most cancers. By setting a stringent cutoff as having a correlation with CS or TEN1 in at least 10 from the 33 cancer types, we discovered both positively (in red) and negatively (in blue) correlated compounds (Figures 7A,B). Several cell growth inhibitors, such as inhibitors for AKT, EGFR, mTOR, and PI3K, were positively correlated with CS score, implying that inhibition of cell growth might induce CTC1 and STN1 expression (Figure 7A). In contrast, chemical compounds affecting the metabolism were potential compounds regulating CTC1 and STN1 negatively. These compounds include the nitric oxide synthase inhibitor, bile acid, and pyruvate dehydrogenase kinase inhibitor (Figure 7A).

### Building a User-Friendly, Open-Access CST Database

**FIGURE 8:**
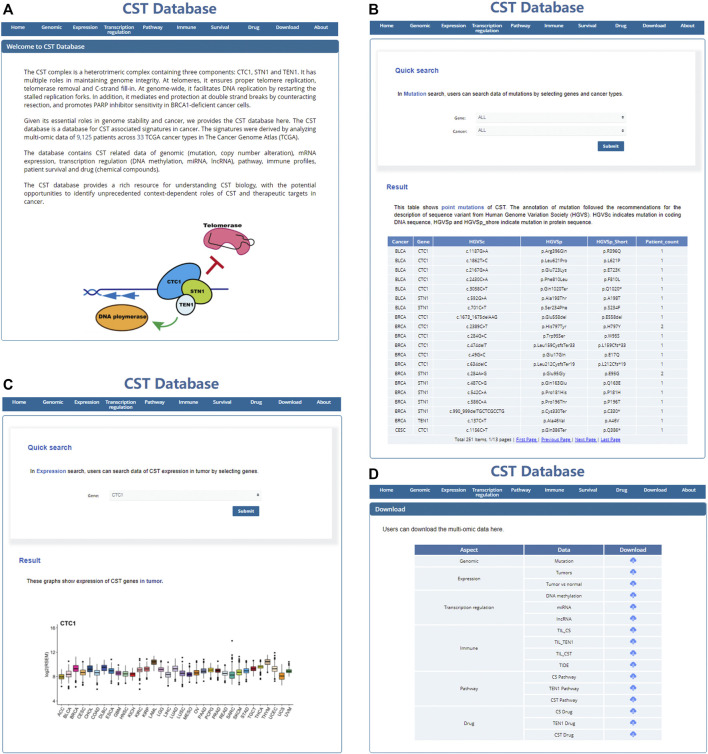
User-friendly, open access CST database. Screenshot of the home page **(A)**, search page **(B,C)**, and download page **(D)** of the CST database.

To make the data available and benefit the research community, we have built a user-friendly, open-access database named CST database (http://bioinfo-sysu.com/CSTDatabase/) (Figure 8A). Users can search and download the multi-omic data (Figures 8B–D). These data repositories of the secondary analysis are allowed to upload and release publicly.

## Discussion

As a key regulator of telomere maintenance and genome integrity, the CST complex has been implicated in the regulation of tumorigenesis. Our systematic study of the CST complex and the online database will serve as a valuable resource for understanding telomere dysfunction in cancer and help for the development of CST targeting therapy. When our work was under review, a similar study analyzing the TCGA cohort was published (Dos Santos et al., 2022). Although we used different bioinformatics tools, we had similar observations of genetic alterations for CST and CST-associated features of survival, immune response, microRNAs, and drugs. Besides, our study provides more detailed analyses on CST expression patterns and their association with immune checkpoint blockade.

Our work suggests that CTC1 and STN1, but not TEN1, are putative tumor suppressors. Supporting this, tumors with high a level of CTC1 and STN1 display low levels of telomerase activity, cancer stemness (an indicator of cancer progression), and genome instability. Moreover, CTC1 and STN1 are predictive of better survival. Consistent with this, knockout of CTC1 enhanced the oncogene c-MYC mRNA level.

Immunotherapy has been approved for treating the most advanced malignancies (Waldman et al., 2020). T cells are the main effector cells of immune checkpoint blockade (Waldman et al., 2020). First, our results show a positive correlation between CS score and CD8 T cells in most cancer types. Second, we find that the efficacy of immunotherapy is not only related to the proportion of CD8 T cells but also related to T cell dysfunction and T cell exclusion, which can be measured by TIDE score (Jiang et al., 2018). The negative correlation between CS score and TIDE suggests that CS score is negatively associated with tumor immune escape. In addition, studies have observed the existence of tertiary lymphoid structures (TLSs) in tumors, and B cells regulate the function of T cells through antigen presentation and antibodies (Cabrita et al., 2020; Helmink et al., 2020). CD8 T cells and B cells have a synergistic effect in tumor immunotherapy (Cabrita et al., 2020). We observed that CS score was also positively correlated with tumor-infiltrating B cells, and CS score may predict the efficacy of immunotherapy. Consistently, we found that CS score was associated with better response to immune checkpoint blockade. In three melanoma studies, CS score was positively correlated with PD-L1 expression. In the IMvigor study, blockage of the TGF pathway was found to enhance the efficacy of immunotherapy (Mariathasan et al., 2018), and we found a positive correlation between the CS score and TGF beta receptor 2 (TGFBR2). These suggest that the mechanisms underlying better response seem to be different.

CST is a conserved protein complex in various eukaryotic cells (Price et al., 2010; Lue, 2018). Although the three components of CST are considered as a whole complex, the separated functions of TEN1 have been found. For example, in *Arabidopsis*, CTC1 and STN1 recruit telomerase, while TEN1 works to release telomerase after telomere elongation (Renfrew et al., 2014). Therefore, it is not unpredictable that individual members could also have different functions or that a subcomplex of CST existed in humans. In this study, we notice that TEN1 is not always co-expressed with CTC1 and STN1. First, tumors have less CTC1/STN1 but more TEN1 expression than their adjacent normal tissues in a majority of cancer types. Second, the correlations with DNA damage and proliferation pathways are opposite between CTC1/STN1 and TEN1. Third, Cox regression demonstrates that CTC1/STN1 expression is positively related to patient survival, while TEN1 is negatively related. These data indicate that human TEN1 may have distinct roles from CTC1/STN1. Indeed, a previous study has suggested that TEN1 has an additional role in regulating genome-wide replication as TEN1 depletion results in a higher level of anaphase bridges (Kasbek et al., 2013). TEN1 may help to resolve replication stress to facilitate cancer cell growth. Moreover, the mutually exclusive pattern of genetic alterations of CST indicates that mutation of any one of the three members is sufficient to change the function of the CST complex. Interestingly, CTC1 and STN1 are largely mutated or deleted, while TEN1 is amplified. This also suggests that TEN1 may have different roles compared to CTC1 and STN1.

Regarding the therapeutic potential of modulating CST expression, CST has been found to promote PARP inhibitor sensitivity in BRCA1-deficient cancer cells (Barazas et al., 2018; Mirman et al., 2018). In the connectivity map analysis, several cell growth inhibitors, such as inhibitors for AKT, EGFR, mTOR, and PI3K, are positively correlated with CS score, suggesting that these cell growth inhibitors might increase CTC1 and STN1 expression. Thus, combined treatment of cell growth inhibitors and PARP inhibitors might efficiently kill BRCA1-deficient cancer cells.

In sum, our systematic analyses provide multi-omic molecular signatures and point to protective functions of the CST complex in cancer. We uncover CST-associated genetic, transcriptomic, DNA methylation, miRNAs, and lncRNA signatures and experimentally validate that CTC1 may be a negative regulator of c-MYC. We also identify CST-related tumorigenesis pathways and immune profiles as well as the correlation with patient survival and potential CST targeting drugs. To make the data available and benefit the research community, we have built a user-friendly, open-access database named CST database (http://bioinfo-sysu.com/CSTDatabase/) for users to search and download data.

## Data Availability

The original contributions presented in the study are included in the article/Supplementary Material, and further inquiries can be directed to the corresponding authors.
