# Supplementary material for: Pan-Cancer Analyses Identify the CTC1-STN1-TEN1 Complex as a Protective Factor and Predictive Biomarker for Immune Checkpoint Blockade in Cancer
Source: Front Genet. 2022 Mar 16;13:859617. doi: 10.3389/fgene.2022.859617 (PMC8966541; doi:10.3389/fgene.2022.859617)
Supplement: Supplementary file 3 [file DataSheet1.PDF]

Supplementary Information

Supplementary Figure legend

Supplementary Figure 1. Mutational Hotspots of CST and Expression of CST

Plots showing mutations of CST genes. Hotspot mutations with > 3 incidents are labeled. Green dot indicate missense mutation. Black dot indicates truncation. Mutations that result in substitutions are indicated by single-letter amino acid code separated by slashes. (B) Boxplots showing expression of individual CST gene in patient samples with no alteration, mutations, amplification and deep deletion of correspondent gene. The differences between any two groups were tested by wilcox rank sum test.

Supplementary Figure 2. CST correlated pathways

(A)The heatmap show normalized enrichment score of significant hallmarks sets of CST score. (B)The heatmap show normalized enrichment score of significant hallmarks sets of CS score (left) and TEN1 expression (right). Each column represents a cancer type and each row represents hallmark set. The Red represents positive normalized enrichment score, and blue represents negative enrichment score. The “*” symbol in cells indicates enrichment was statistical significant. Unsupervised clustering used euclidean distance metric with complete linkage. The function of the significant hallmark sets are annotated in different colors.
